# Coping Styles and Alcohol Dependence among Homeless People

**DOI:** 10.1371/journal.pone.0162381

**Published:** 2016-09-06

**Authors:** Cezary Opalach, Jerzy Romaszko, Marcin Jaracz, Robert Kuchta, Alina Borkowska, Adam Buciński

**Affiliations:** 1 Faculty of Theology, University of Warmia and Mazury in Olsztyn, ul. Kard. Hozjusza 15, 11–041 Olsztyn, Poland; 2 Department of Family Medicine, University of Warmia and Mazury in Olsztyn, ul. Warszawska 30, 10–082 Olsztyn, Poland; 3 Department of Clinical Neuropsychology, Ludwik Rydygier Collegium Medicum in Bydgoszcz, Nicolaus Copernicus University in Toruń, ul. M. Curie Skłodowskiej 9, 85–094 Bydgoszcz, Poland; 4 Municipal Social Welfare Centre in Olsztyn, ul. Towarowa 18, 10–417 Olsztyn,Poland; 5 Department of Biopharmacy, Ludwik Rydygier Collegium Medicum in Bydgoszcz, Nicolaus Copernicus University in Toruń, ul. A. Jurasza 2, 85–089 Bydgoszcz, Poland; Nathan S Kline Institute, UNITED STATES

## Abstract

**Background and Objectives:**

The ways in which homeless individuals cope with stress may differ from those relied upon by the members of the general population and these differences may either be the result or the cause of their living conditions. The aim of the study was to determine the preferred coping style among the homeless and its relationship with alcohol dependence.

**Methods:**

The study included 78 homeless individuals and involved the collection of demographic, sociological, psychological and medical data from each participant. Coping styles relied upon when dealing with stressful situations were assessed using a Polish adaptation of the Coping Inventory for Stressful Situations. Alcohol dependence was assessed using the Michigan Alcoholism Screening Test (MAST) and a quantitative analysis of alcohol consumption.

**Results:**

Men accounted for 91.93% of the study population. Nearly 75% of the subjects met the alcohol dependence criterion. Significant relationships were observed between the individual's age, preferred coping style and alcohol consumption level. As an individual’s age increased, the use of emotion-oriented coping styles decreased, while an increase in alcohol consumption was associated with a more frequent use of emotion- and avoidance-oriented strategies.

**Conclusions:**

The findings of this study, similarly to those of many other studies of homeless individuals but investigating other areas (e.g. epidemiology of tuberculosis and traumatic injuries), are an exaggerated representation of associations observed in the general population. The results describe a group of people living on the margins of the society, often suffering from extremely advanced alcoholism, with clear evident psychodegradation. The presence of specific ways of coping with stress related to excessive alcohol consumption in this group of individuals may interfere with active participation in support programmes provided for the homeless and may further exacerbate their problems.

## Introduction

Homeless people living on the margins of industrialised European societies are perceived as a social and economic menace, as well as a public-health risk. It is relatively rarely recognised that this social group is heterogenous. This group comprises chronically homeless individuals and individuals whose homeless periods are interspersed by periods of being taken in by their relatives or friends or periods of temporary housing. The causes of homelessness vary and include structural factors, such as poverty, and individual factors, such as substance dependence, criminal offences or familial conflicts, however, in any single individual, the cause may be the result of a combination of these factors [1]. A considerable numeric predominance of men, a high or very high percentage of individuals dependent on alcohol and/or drugs, a high prevalence of mental health problems, and a high rate of offences are some of the typical characteristics of homeless populations [2–5]. The results of a meta-analysis performed by Fazel et al. show that the prevalence of alcohol dependence in the homeless population is 8.5–58.1% [5]. However, the data included in the analysis concerned only men from Western countries. In Europe, e.g. in the Danish homeless population, 38.4% of men and 36.9% of women were dependent on alcohol, and a previous study (covering the years 1997–1999) performed by Salize et al. conducted in Mannheim, Germany, determined that 63.7% of the homeless individuals were either dependent on alcohol or displayed a risky pattern of drinking [6, 7]. Braun et al. analysed data from Hungary and concluded that the most common disorders encountered among homeless men were personality disorders and alcohol dependence [8]. Much lower rates were determined by Morikawa et al. based on data obtained in Tokyo, Japan, where only 15% of the subjects were found to be dependent on alcohol, with men accounting for as many as 93.8% of the study population [9].

There is, however, a fundamental difference, most likely rooted in the cultural background: homelessness is 60 times more prevalent in New York City than in Tokyo [10]. Another notable finding is the higher level of education among the homeless in Japan [11]. One of the rather scarce publications from Africa reports on an Ethiopian study (Addis Ababa) according to which 60% of homeless individuals declared risky drinking or alcohol dependence, with men accounting 89.9% of the study population [12]. The specific type of psychoactive substance is of minor significance and is largely determined by the local environment. This explains why drug dependence is more commonly reported by American authors, while alcohol dependence prevails in European publications.

Stress in general can be defined as a non-specific response of the body to noxious stimuli called "stressors" [13]. Psychological stress is a particular relationship between the person and the environment that is appraised by the person as taxing or exceeding his or her resources and endangering his or her well-being [14, 15].

A significant role in stress appraisal is played by cognitive appraisal which is responsible for interpreting and classifying a given stimulus as a threat, as a result of which the stimulus is perceived by the person as a stressor [14]. Cognitive appraisal involves two processes: primary and secondary appraisal. *Primary appraisal* is the process through which the person interprets and classifies his or her relationship with the environment as harm/loss, e.g. loss of someone close, damage to social esteem, threats, where harms or losses are anticipated, or challenges, where harm or loss is accompanied by anticipation of gain. *Secondary appraisal* refers to the process of defining what can be done to remove the stressor or alleviate its impact or, if the situation has been classified as a challenge, what can be done to gain from it. All these activities are referred to as ways of coping with a stressful situation, and attempts to operationalise them have been made by many authors, including Norman S. Endler and James D.A. Parker, who proposed to refer to them as coping styles [16].

According to Lazarus, the way an individual copes with stress does not only have a short-term effect on how they are feeling but, in the long run, also affects their somatic health and social functioning. It is interesting why, in some people, stressful situations lead to life crises (e.g. homelessness), while in others they do not. Within the same population of homeless people, the quality of coping with stress affects the level of mental health and consumption of psychoactive substances, which translates into the chances of transitioning out of homelessness [1].

Current data show that stress related disorders (e.g. depression) appear as a common problem among homeless subjects and may interfere with functioning, mostly with securing stable housing, obtaining employment, and health services. Victimization and exposure to parent problems in childhood as well as intensity of current psychological stressors are considered as important risk factors for the occurrence of psychiatric disturbances and alcohol abuse in the homeless. These factors may also be related to the development of specific stress coping behavior related to poor social resources [17].

The purpose of this study was to assess the stress coping styles in relation to alcohol consumption and alcohol dependence in Polish homelessness.

## Material and Methods

### Study procedure

This paper is an element of a research project carried out by the Department of Family Medicine, University of Warmia and Mazury in Olsztyn, Poland. The project was entitled "An evaluation of the health status of the homeless population" and involved the investigation of homeless individuals between December 2013 and November 2014 in Olsztyn, Poland. Olsztyn has a population of approximately 175 thousand that—according to the 2014 data published by the Regional Centre for Social Policy—includes 153 homeless people falling within the European Typology on Homelessness and Housing Exclusion (ETHOS) operational categories 1, 2 and 3.1 (i.e. people staying at homeless hostels, overnight shelters or in public spaces) [18].

Participation in the study was entirely voluntary and no compensation was provided for participation. The subjects had the right to refuse to answer any of the questions without stating the reason. Each subject first received detailed written information on the purpose of the study and then provided written consent to participate in the study. Visually impaired and/or illiterate subjects were read the subject information by the investigator. The study was conducted personally by the authors of this publication at the Shelter for Homeless People in Olsztyn, Poland. Roofless homeless individuals were invited by their social workers to the shelter. Those who were likely to be under the influence of alcohol or other psychoactive substances at the moment of conducting the study assessments were excluded from the study (these individuals were re-invited to attend at a later date). The study protocol was approved by the Bioethics Committee of the University of Warmia and Mazury in Olsztyn, Poland. The entity responsible for the storage, coding and future use of the medical records was the Department of Family Medicine at the University of Warmia and Mazury in Olsztyn, Poland. The authors declare no conflicts of interest in relation to this article.

### Subjects

The study population comprised 78 homeless individuals. (ETHOS operational categories 1.1, 2.1 and 3.1). A total of 48 subjects (61.54%) made use of overnight shelters (ETHOS operational category 2.1), 24 subjects stayed at homeless hostels (ETHOS operational category 3.1) and 6 subjects lived in public spaces (ETHOS operational category 1.1). It should be emphasised here that this division is quite artificial because a homeless individual who was staying at an overnight shelter on the day of the study assessment could become a roofless individual the very next day. In our database, 42 subjects (53.85%) were individuals who periodically lived in public spaces (ETHOS operational category 1.1). The study population was dominated by individuals who had been homeless for a long time, with as many as 55 subjects (70.51%) having been homeless for more than 3 years, and 22 subjects (28.21%) for more than 10 years. The mean age was 55.36 (SD 9.81) years, 8.97% [7/78] were female and 91.03% [71/78] were male. Their level of education was quite low, with an average school attendance level of 10.47 (SD 3.08) years and 23.08% (18/78) of the participants having repeated a year at least once. Most of them (93,59% [73/78]) were single and mainly divorced (57.69% [45/78]).

### Stress coping

The coping styles preferred by the homeless individuals when dealing with stressful situations were determined using the Polish adaptation of the *Coping Inventory for Stressful Situations* (CISS) developed by N.S. Endler and J.D.A. Parker [19]. The CISS contains 48 items describing various behaviours in stressful situations and distinguishes the following types of coping styles:

1. Task-oriented coping, where a major role is played by cognition which allows the individual to focus on the task and find a solution to the problem.
2. Emotion-oriented coping, which focuses on the person's own emotions in order to reduce the emotional tension caused by the stressor. This coping style is therefore focused on the individual rather than being directed at the problem.
3. Avoidance-oriented coping, which refers to a strategy aimed at avoiding thoughts and emotions associated with a stressful situation. Avoidance may take the form of:
  - Distraction with other activities (such as binge eating, watching television).
  - Social diversion.

Each of the 48 items is rated on a five-point Likert-type rating scale ranging from 1 to 5. The potential range of scores on each of the three scales (Task, Emotion, Avoidance) is from 16 to 80. The possible range for the Distraction subscale is from 8 to 40 and for Social Diversion the range is from 5 to 25.

### Alcohol use

Alcohol dependence, was assessed using the Michigan Alcoholism Screening Test (MAST) and a quantitative analysis of alcohol consumption by the type of alcoholic drink (beer, wine, liquor, non-beverage alcohol). Persons with a MAST score of at least 5 were considered to be alcohol-dependent [20]. The type and amount of alcohol consumed was converted in our study using conversion tables and expressed it in the standard units of alcohol per week. 10 g were adopted as the standard unit of alcohol (given the fact that the definition of a standard unit of alcohol varies from country to country, the variant accepted by the European Commission and the WHO was adopted in the present study) [21].

### Statistical analysis

A statistical analysis was performed using software manufactured by StatSoft, Inc. (2014), STATISTICA (data analysis software system), version 12. www.statsoft.com. The study variables were characterised using descriptive statistics (arithmetic mean, standard deviation, minimum, maximum). Parametric tests were used for variables meeting the assumptions of normality and homogeneity of variance. Otherwise, suitable non-parametric tests were used. The assumption of normality was tested using the Shapiro-Wilk test, and the assumption of homogeneity of variance was tested using the Levene's test. A statistical significance level of p = 0.05 was adopted. The internal consistency of the CISS subscales was measured with Cronbach's alpha.

## Results

### Alcohol

The results have shown the mean weekly alcohol consumption levels by homeless individuals expressed in standard portions to be 30.77 SD 58.22 (see Table 1).

**Table 1:** The structure of alcohol consumption by homeless individuals.

Alcohol (types)	Mean*	SD
Alcohol portions, total	30.77	58.22
Vodka, portions	13.32	42.13
Wine, portions	5.10	16.20
Beer, portions	12.65	31.42

*N = 75

The MAST criterion of alcohol dependence (a total score of 5 or more) was met in 74.64%(59/78) of the subjects with a further 7.69%(6/68) of the subjects being considered as possibly dependent (a total score of 4). Only 16.67% (13/78) of the subjects did not meet the dependence criterion (a total score of 3 or less). Occasional consumption of non-beverage alcohol was declared by 12 subjects (15.38%).

### CISS

The analysis of the study questionnaires CISS allowed to characterise the homeless individuals in terms of coping styles. The results of this analysis are given in Table 2.

**Table 2:** Mean scores in the CISS.

Coping style	Mean*	SD	Minimum	Maximum
Task	52.49	9.92	31	76
Emotion	46.14	10.84	21	69
Avoidance	46.44	9.47	16	73
Distraction	22.32	5.90	8	37
Social Diversion	14.79	3.95	5	25

*N = 78

Based on the statistics in Table 2 it may be concluded that the homeless individuals in the present study, in the main scales, achieved the highest score on the task-oriented coping scale (52.49) and the lowest score on the emotion-oriented coping scale (46.14). In light of the fact that the mean score for the main scales is 48, it is clear that the subjects exceeded this threshold only for the task-oriented coping scale and did not so for avoidance-oriented and emotion-oriented coping scales.

Further analysis of the main scales demonstrates that the most homogenic subset of results consists of the results achieved on the avoidance-oriented scale (where SD was 9.47), while the greatest dispersion of the results is observed for the emotion-oriented scale (as evidenced by the SD value of 10.84).

The greatest consistency of results was, however, achieved on the social diversion subscale (with an SD of 3.95) and on the distraction subscale (with an SD of 5.90). A closer look at the data and the mean scores on the subscales reveals that they are very similar to the results reported by the group of alcoholics that were investigated during the normalisation study of the Polish version of the CISS [19].

The CISS results in the present study were compared with various elements concerning of lifestyle and environment of homeless people. Table 3 lists only those of them for which statistically significant associations were found.

**Table 3:** Spearman correlations of the results for each of the CISS scales and subscales with age and with alcohol consumption.

Element of the life environment	CISS scales and subscales (r; n; p)		
	Task	Emotion	Avoidance
Age	-0.069; 78; 0.550	-0.360; 78; 0.001	-0.049; 78; 0.137
Beer	0.111; 78; 0.342	0.237; 78; 0.041	0.274; 75; 0.018
Vodka	0.073; 75; 0.533	0.332; 75; 0.004	0.209; 75; 0.073
Alcohol, total	-0.006; 75; 0.960	0.416; 75; 0.000	0.266; 75; 0.021

The results of the analysis provided in Table 3 show that the use of emotion-oriented coping strategies by the homeless correlates negatively with age (r = –0.360, p = 0.001). The younger subpopulation of the homeless individuals was found to cope with stressors by focusing on emotions, while the older subpopulation undertook more constructive activities.

Table 3 shows that the degree of reliance on emotion-oriented coping is directly proportional to the level of alcohol consumption.

The data obtained using the CISS also allowed to determine the dominant coping style in each subject. If the score on one scale is at least two sten scores higher than the scores on each of the two remaining scales, the coping style represented in the scale is considered dominant [19]. Two other patterns found in the results illustrated a situation in which two styles dominated the third one but did not differ between themselves and a situation in which the differences between all the three styles were not statistically significant. The former situation was referred in the present study as "mixed styles" and to the latter as a "no dominant style", while a situation of significant dominance of one style over the two others was referred to as "the dominant style". Detailed results of the distribution of these categories are given in Table 4.

**Table 4:** Distribution of coping style categories.

**Dominant styles**	**N**	**%**
Task	6	7.69
Emotion	9	11.54
Avoidance	19	23.08
No dominant style	20	25.64
Mixed styles	24	30.77
**Mixed styles**	**N**	**%**
Emotion/Avoidance	16	20.51
Task/Avoidance	5	6.41
Task/Emotion	3	3.85
**Dominant styles (mixed styles included)**	**N**	**%**
Avoidance	40	51.28
Emotion	28	35.90
Task	14	17.95

The data provided in Table 4 indicate that the most common way of coping with stress relied upon by homeless individuals (24 subjects, i.e. 30.77% of the study population) involves a mixture of two styles which dominate the other style. According to Table 4, a combination of emotion- and avoidance-oriented styles is the most common one (16 subjects, i.e. 20.51% of the study population) and a combination of task- and emotion-oriented styles is the least common one (3 subjects, i.e. 3.85% of the study population). It is also apparent that the avoidance-oriented style was involved in 87.5% of the situations where two styles were used in combination (21 in 24 subjects). Further analysis of the results shows that the second most common category to "mixed styles" is the "no dominant style" category. It means that 20 subjects, i.e. 25.64% of the population, rely alternately on all three coping styles when faced with stressful situations.

Lastly, in situations in which reliance on only one style predominates, it is most commonly the avoidance-oriented style (19 subjects, i.e. 23.08% of the study population), while the least common was the task-oriented style (6 subjects, i.e. 7.69% of the study population), as confirmed by the association indicated in Table 4, according to which homeless individuals most commonly use the avoidance-oriented style when faced with problem situations.

In situations where one style predominated or two co-dominant styles were found, the avoidance-oriented coping style was the style most commonly relied upon (40 subjects, i.e. 51.28% of the study population), while the task-oriented coping style was the style least commonly relied upon (14 subjects, i.e. 17.95% of the study population).

The next comparison (Table 5) shows associations between the prevalence of specific coping styles, the mean age of the subjects who preferred that specific style and the number of subjects who were alcohol-dependent in that group.

**Table 5:** The prevalence of specific coping styles, the mean age of the subjects who preferred a specific style and who were dependent on alcohol.

Element of the environment	Coping style		
	Avoidance	Emotion	Task
Number of subjects relying upon the style	40	28	14
Percentage of subjects relying upon the style	51.28	35.90	17.95
Mean age of subjects relying upon the style (years)	55.90	53.75	59.93
Number of subjects dependent on alcohol	30	24	10
Percentage of subjects dependent on alcohol	76.92	85.71	71.42

The results in Table 5 show that the older subjects (59.93 years, SD 7.26) are more likely to try to overcome stressful situations by cognitive transformation or changing the situation (task-oriented coping), while the younger subjects (53.75 years, SD 10.61) would commonly resort to wishful thinking and fantasising (p = 0.029). The mean age of subjects dealing with a stressful situation by avoiding thoughts and emotions associated with the situation (avoidance-oriented coping) is 55.90 years (SD 10.68).

The percentage of alcohol-dependent subjects was found to be 76.92% in the group relying upon avoidance-oriented coping and as much as 85.71% in the group relying on emotion-oriented coping. In the study population, 16 subjects relied upon a mixed, emotion/avoidance, coping style and 81.25% of them met the criterion of alcohol dependence.

The next step of the analysis involved an assessment of the reliability of the CISS scales. Cronbach's alpha values for the Task, Emotion and Avoidance scales and the Distraction and Social Diversion subscales were: 0.854, 0.851, 0.777, 0.723 and 0.655, respectively.

## Discussion

Results of psychological studies in this particular subpopulation should be analysed in the sociological context. As K. Fergusson and S. Thomason have pointed out, such analyses should take into account the considerable heterogenicity of this group of people [22, 23]. Ethnic minorities in the European data on homeless people may account for up to 50% (France), and are always several times higher than their value in the general population of the country in question [24]. In American reports, African Americans often account for 70–90% of homeless people [25, 26]. The situation in Poland is, however, different [24]. The Polish society is quite homogenic, and national ethnic minorities account for a mere 1.55% of the general population, with the most numerous minorities (but entirely insignificant from the viewpoint of the present analysis) being Germans, Lithuanians and Belarusians [27]. For instance, African Americans account for a mere 5% of all immigrants, i.e. about 5500 people nationwide. Because the homeless are, in a sense, a product of the society in which they live, the population in the data from the present study was quite homogenic. All the subjects in the present study were Caucasian.

Although males largely predominated (91.03%) in the study population, this was consistent with literature and government data. In the region where the present study was conducted (Olsztyn, Poland), according to the Regional Centre for Social Policy, the male predominance among the homeless amounted to 91.50% in 2014 and 87.29% in 2015. This phenomenon is also observed in other European countries. The FEANTSA report of 2014 estimated the percentage of males at about 62–87% depending on the country, with the highest value being reported for Italy (87%) and the lowest for France and Sweden (62% and 64%, respectively). In Russia, the percentage of males among the homeless is estimated at 85–90% [28]. The percentage of males in the United States is reported at 63% [24]. A vast majority of homeless individuals, often up to 90%, are people living alone (93.59% in the present study) [29–31]. The mean age of homeless individuals in the study population was 55.36 years, which was quite typical of the values reported in similar studies for Central European countries [24]. While they most commonly deal with slightly younger populations, American and Western European authors also emphasise the phenomenon of ageing in their homeless populations [5, 32, 33].

The analysis of the data obtained in the present study showed that a considerable number of homeless individuals preferred avoidance-oriented coping style, emotion-oriented coping style or both. Lazarus distinguished only two coping styles (emotion-focused and problem-focused) and what he defined as emotion-focused coping is similar to emotion- and avoidance-oriented coping styles in the CISS. Studies have shown emotion-oriented coping style to be associated with a poorer health and to be less effective in the long run, as it does not lead to solving the problem [34]. Emotion-oriented coping style is superior only in situations where we cannot realistically influence the situation. Studies have also shown that task-oriented coping style is used by those who simply believe that they can affect the situation they found themselves in [35]. This suggests that homeless individuals could not be strongly convinced that they are able to influence their life circumstances a finding corroborated by other studies [36].

When analysing the ways homeless people cope with stress it should be kept in mind that 3/4 of the subjects in the present study (74.64%) were dependent on alcohol (MAST scores of ≥5). This result probably affects all the associations that have been identified. Chronic and habitual alcoholism induces numerous organic changes in the central nervous system. These changes range from cerebral atrophy, through changes in the frontal lobes (decreased frontal lobe density of about 15–23% and decreased frontal lobe volume), injuries affecting the limbic system structures, particularly the hippocampus), hypothalamus and mamillary bodies (especially in correlation with vitamin B deficiency in Korsakoff syndrome), and very typical (although less important for our considerations) cerebellar injuries [37–44]. In the present material, the mean weekly consumption of alcohol (Table 1) was almost twice as high when compared to the national average, with mainly vodka and beer being preferred, which was in contrast to the general population, in which beer, followed by vodka was preferred [45].

Furthermore, the data in the present study suggest a relationship between alcohol dependence and reliance upon avoidance- and emotion-oriented coping styles (Tables 5 and 3). The reliance upon the avoidance-oriented coping style by half of the study population when trying to overcome stressful situations and upon the emotion-oriented coping style by 35.90% of the subjects with 76.92–85.71% of the subjects in both these groups being alcohol-dependent, suggests regulation of emotions typical of addiction with a narrow repertoire of problem-solving skills, which are related to the so-called executive functions coordinated by the prefrontal cortex. Hence the obvious association with the consequences of frontal cortex injuries, which are typical of alcohol dependence [46]. Of note is the extensive similarity between the findings of the present study and those obtained for the group of alcoholics investigated during normalisation studies of the Polish version of the CISS. The values determined in the present study are almost identical, although there is one significant difference. While the homeless individuals in the present study scored an average of 46.14 on the emotion-oriented coping scale, the mean score obtained for the normalisation group alcoholics was 54.12 (p = 0.000). Under normal conditions, individuals dependent on alcohol experience a certain inner conflict between the need to drink and the awareness that their drinking needs to be limited [47]. In extreme cases it may, however, be justified to assume that individuals with a low level of emotional intelligence are unable to reflect upon, and therefore focus on, the emotions they experience, in contrast to what happens with emotion-oriented coping style. This self-reflection capability seems to be present in the normalisation group, i.e. in the group of individuals who presented to a rehabilitation clinic looking for help [19].

T.B. Horvath has suggested that alcoholics suffer from disturbances of abstract thinking with 95% of them experiencing disturbances of working memory, 85% displaying behavioural disorders, 81% reporting depressed mood, 76% experiencing disorientation to time, 61% disorientation to space, and 35% emotional lability [48].

Individuals with alcohol dependence, when faced with stressful situations and the related unpleasant emotional states, adopt a mechanism of emotional regulation that is typical of addiction, and make every effort to immediately soothe these disturbing emotions with alcohol rather than attempting to alter the stressful circumstances. Three-factor feedback therefore develops, where stressful situations contribute to the generation of unpleasant emotions which are subsequently neutralised by alcohol, which in turn increases the severity of alcohol dependence and the tendency to reach for alcohol in the future, when faced with difficult situations again. This type of reaction corresponds to the emotion-oriented coping style, where the individual-rather than facing up to the difficulties and dealing with them in a creative way-focuses on his or her own emotions (i.e. adopts a self-oriented style) and uses this approach to reduce the emotional tension caused by the stressor. This phenomenon is responsible, among other things, for facilitating frequent use of analgesics by dependent individuals [49].

The issue of high mortality rates among homeless people, especially in the younger age groups, cannot be overlooked here. The average 30-year-old homeless person has a life expectancy of only 11 years [50]. Many studies have demonstrated a correlation between mortality and alcohol consumption (the so-called U-curve) [51]. The present study has revealed a statistically significant correlation between age and alcohol consumption (younger homeless individuals drink more). It is therefore quite likely that the excess mortality among young homeless people widely reported in the literature and the high alcohol consumption in this group are interrelated. These facts suggest a possible influence of the natural selection mechanism in our findings. Younger homeless individuals who drink more alcohol, i.e. those who use emotion-oriented coping strategies, are at a higher risk of death. Prolonged homelessness may also lead to the accumulation of risk factors for premature death (somatic illnesses, but also victimisation). Such resources as social support and access to material help and healthcare are also diminished [1]. A considerably elevated risk of premature death is also observed in homeless individuals addicted to psychoactive substances. In addition to the risk of overdose, other factors that come into play are the lack of self-care in terms of personal health or sleeping rough, which may lead to death due to hypothermia in the winter period [52, 53].

The present study has also shown a correlation between increasing age and less frequent reliance upon emotion oriented coping styles among the homeless (Table 3).

As already mentioned, alcohol abuse probably affects all the associations identified in the present study. Alcohol abuse leads to serious disturbances of memory function (as discussed before). This explains the associations observed in the present study, implying that half of the study population relies on the avoidance-oriented coping style as the most common tool in fighting the stress, that involves avoiding thoughts and emotions associated with the problem situation. Subsequently, the results of the present study seem to confirm that the subjects were generally incapable of creative thinking in difficult situations, as the deficiencies in their working memory made it impossible. On the other hand, because they were dependent on alcohol (or displayed some other types of intellectual deficit), they used the classical escape mechanism when faced with a problem, escaping into the world of dependence in the hope that the problem would spontaneously resolve.

The results of the present study are another piece in the complex jigsaw puzzle reflecting the image of the homeless person. An image, without which the development of support programmes are doomed to failure. Two approaches clash when developing programmes of transitioning out of homelessness: the behavioural approach, according to which the homeless individual can only be helped in return for something (giving up an addiction, starting employment, etc.), and the other approach, according to which any human being deserves help, without having to meet any additional conditions. Examples of the latter approach are numerous housing first (HF) programmes that are in place in Denmark and Finland, for instance. HF programmes are expensive and sometimes generate opposing emotions in society (someone gets something for free), but they are also evidently more effective [54]. In Poland, provision of assistance to the homeless is a statutory obligation of local governments at the level of municipalities. This obligation, however, makes an a priori assumption about collaboration on the part of the homeless person in solving their own life problems. In other words, it makes assistance conditional upon the demonstration of some initiative on the part of the homeless person. In light of the results of the present study, however, such an initiative would be hard to expect.

### Limitations

Conducting research amongst the homeless population can be a difficult task. Issues such as a lack of interest, simulation or dissimulation must always be taken into account. Retrospective collection of data on living conditions, duration of homelessness or alcohol consumption, to name but a few, may therefore be burdened with error. The authors of the present study were aware of those factors when they were designing the study, so they tried to select specific questions for our questionnaire and specyfic specific psychological tests to minimise this risk. To collect the data on alcohol consumption, a simple table was used in which the subjects marked how many bottles of beer, wine, vodka or non-beverage alcohol they drank weekly, on average. Every doubt was explained. The resulting data were converted into standard units of alcohol. The CISS questionnaire, however simple to administer, requires that the subjects fully understand the questions. Some of the questions (particularly those about acting out on stress by going shopping or eating excessive amounts of one's favourite food) do not seem to be appropriate for the study population. The entirety of the results is, however, quite consistent. Cronbach's alpha values for the Task and Emotion scales and for the Avoidance subscale were high and equalled 0.854, 0.851 and 0.777, respectively. The present study was also limited by the small size of the study population and the single-centre nature of the study. It should, however, be taken into consideration that over 50% of the homeless population of Olsztyn was assessed, and Olsztyn has a population of 175 thousand (the capital of a region). By taking into account the seasonal migration to large city agglomerations typical of chronically homeless individuals (who stand better chances of surviving, especially in the winter, in those agglomerations), it is highly unlikely for a preselection basis to be present in the data. It is beyond any doubt that further studies of coping with stress by the homeless conducted in other centres will be useful in gaining a better understanding of this topic.

## Conclusions

When attempting to deal with stressful situations, homeless individuals most preferably rely upon avoidance-oriented and emotion-oriented coping styles, while task-orientated coping was the style least relied upon. The frequency of using emotion-oriented coping strategies decreases with age, while alcohol consumption is associated with the tendency to rely upon the poorly effective emotion and avoidance-oriented strategies. It seems that the findings of the present study, just like those of many other studies conducted in homeless individuals but investigating other areas (e.g. epidemiology of tuberculosis and traumatic injuries), are an exaggerated representation of associations observed in the general population. The present results describe a group of people living on the margin of the society, often suffering from extremely advanced alcoholism, with clearly evident psychodegradation, This suggests the presence of organic brain abnormalities and marked intellectual deficits and encourages further investigation among this group of individuals using other techniques, such as functional MRI and/or tests of cognitive function, tests of memory function. The presence of specific ways of coping with stress related to excessive alcohol consumption in this group of individuals may interfere with active participation in support programmes provided for the homeless and may further exacerbate their problems.
